# Foraging activity of sperm whales (*Physeter macrocephalus*) off the east coast of New Zealand

**DOI:** 10.1038/s41598-019-48417-5

**Published:** 2019-08-21

**Authors:** Giacomo Giorli, Kimberly T. Goetz

**Affiliations:** Marine Mammal Laboratory, Alaska Fisheries Science Center, National Marine Fisheries Service, National Oceanic and Atmospheric Administration, 7600 Sand Point Way N.E., Seattle, Washington 98115-6349 USA

**Keywords:** Marine mammals, Marine biology

## Abstract

The occurrence and distribution of sperm whales in New Zealand waters is mainly known from whaling records or opportunistic sightings by the public and a systematic estimation of the abundance and distribution has never been conducted. In this study, we investigated the foraging activity and occurrence of sperm whales off the Eastern coast of New Zealand using passive acoustic monitoring techniques. Three acoustic recorders were moored to the ocean floor at different locations on the east side of the North and South Island to collect passive acoustic data from June 2016 until August 2017. A total of 53,823 echolocation click trains were recorded and analyzed to understand the spatial and temporal variation of sperm whale foraging activity. No difference in the foraging activity was found between night-time and day-time periods at any of the locations. Click train detections increased toward the south, suggesting increased foraging activity near Kaikoura. At each station, sperm whale foraging activity varied by month.

## Introduction

The sperm whale (*Physeter macrocephalus*) is an odontocete, or toothed whale, with a distribution that spans from the ice-edge of both hemispheres to the equator^1^. The conservation status of sperm whales is listed as vulnerable by the IUCN red list^2^ and as “data deficient” by the New Zealand threat classification system^3^. Like other odontocetes, sperm whales hunt using biosonar to detect and range prey in the deep ocean^4^.

Data on the abundance, occurrence, distribution, and foraging behavior of sperm whales in the New Zealand Exclusive Economic Zone (EEZ) are sparse or lacking. Most of the information available are from historic whaling records, opportunistic sightings reported by the public or studies conducted in a known “hot spot” area offshore of the Kaikoura peninsula. Using marine survey data from the Marine Department and catch and aerial survey data from whaling companies, Gaskin^5^ found that sperm whale occurrence in the Cook Strait area varied temporally, peaking between December and April. Other studies have shown that both male and female, mature and immature sperm whales occur in temperate latitudes east of New Zealand to longitude 150° W^6^. In addition, male sperm whales decreased towards the south and virtually no females were found south of 50° S^7^. Some males were found to spend the winter around the Chatham Islands^7^ and solitary sperm whales were reported offshore of Fiordland with observation made from a Japanese long-liner^8^.

Seasonal and spatial occurrence of beach-cast animals showed that sperm whales stranded on both the North and South Island, with no apparent seasonality^9^. A mass stranding event of 72 sperm whales at Muriwai beach in October 1974, was comprised of 54 females, 17 immature males and a bull, providing important information onsocial structure^10^.

In New Zealand, sperm whales have been studied extensively around the Kaikoura peninsula. Between 1988 and 1993, an estimated abundance of 60–108 sperm whales was calculated from 86 sightings in this area and an examination of seasonal residency showed that half the whales were present more than one season^11^. In a similar study carried out between 1990 and 1998, sperm whale abundance around Kaikoura was not found to vary seasonally, but differences between their summer and winter distribution were evident^12^.

Passive acoustic monitoring revealed the occurrence and foraging behavior of sperm whales in other parts of the world such as the Bahamas^13^ and the Pacific Ocean^14–16^. The goal of this study was to examine the foraging activity of sperm whales at three distinct locations along the east coast of New Zealand using passive acoustic monitoring. Foraging activity is defined as the temporal rate of occurrence of sperm whale echolocation signals detected by our recorders in the deep sea. Sequential click detections, hereafter referred to as “click trains”, are in fact associated with the search phase of sperm whale foraging dives^17^. This paper presents results from the first long-term (~15 months) acoustic monitoring study of the temporal and spatial foraging activity of sperm whales in the New Zealand EEZ.

## Results

**Figure 1:**
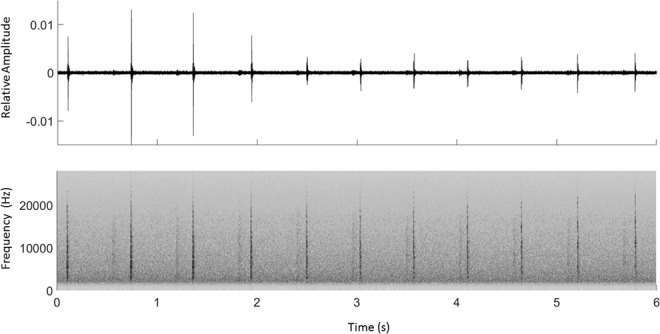
Echolocation click train from a sperm whale recorded at the Castlepoint location. Top panel shows the signals in the time domain. Bottom panel shows a spectrogram of the click train.

The three AMARs collected ~7 TB of data, resulting in a total of 53,823 sperm whale click train detections (22,737 in Kaikoura; 17,622 in Palliser; 13,464 in Castlepoint; an example is shown in Fig. 1) with a precision P of 0.83, a recall R of 0.92 (F-score = 0.84).

**Figure 2:**
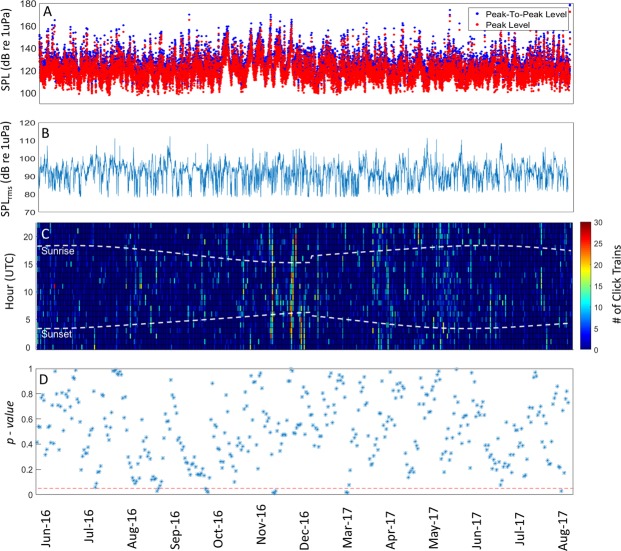
Peak-to-Peak (blue) and Peak (red) Received Levels of the sperm whales click trains detected (panel A); SPL_rms_ Level in the band between 2 and 20 kHz (panel B); Number of click trains detected per hour per day (panel C) and results of the comparison of the number of click trains detected during day-time vs night-time over a 9-day moving window (panel D; one-way ANOVA p-values) for the Castlepoint station. The white dashed lines in panel C indicate sunset and sunrise times. The red dashed line in panel D indicates the 0.05 significance level of the test. P-values below the red dashed line indicate times when night-time and day-time foraging rates were statistically different. No data were collected between 21^st^ December 2016 and 23^rd^ February 2017.

**Figure 3:**
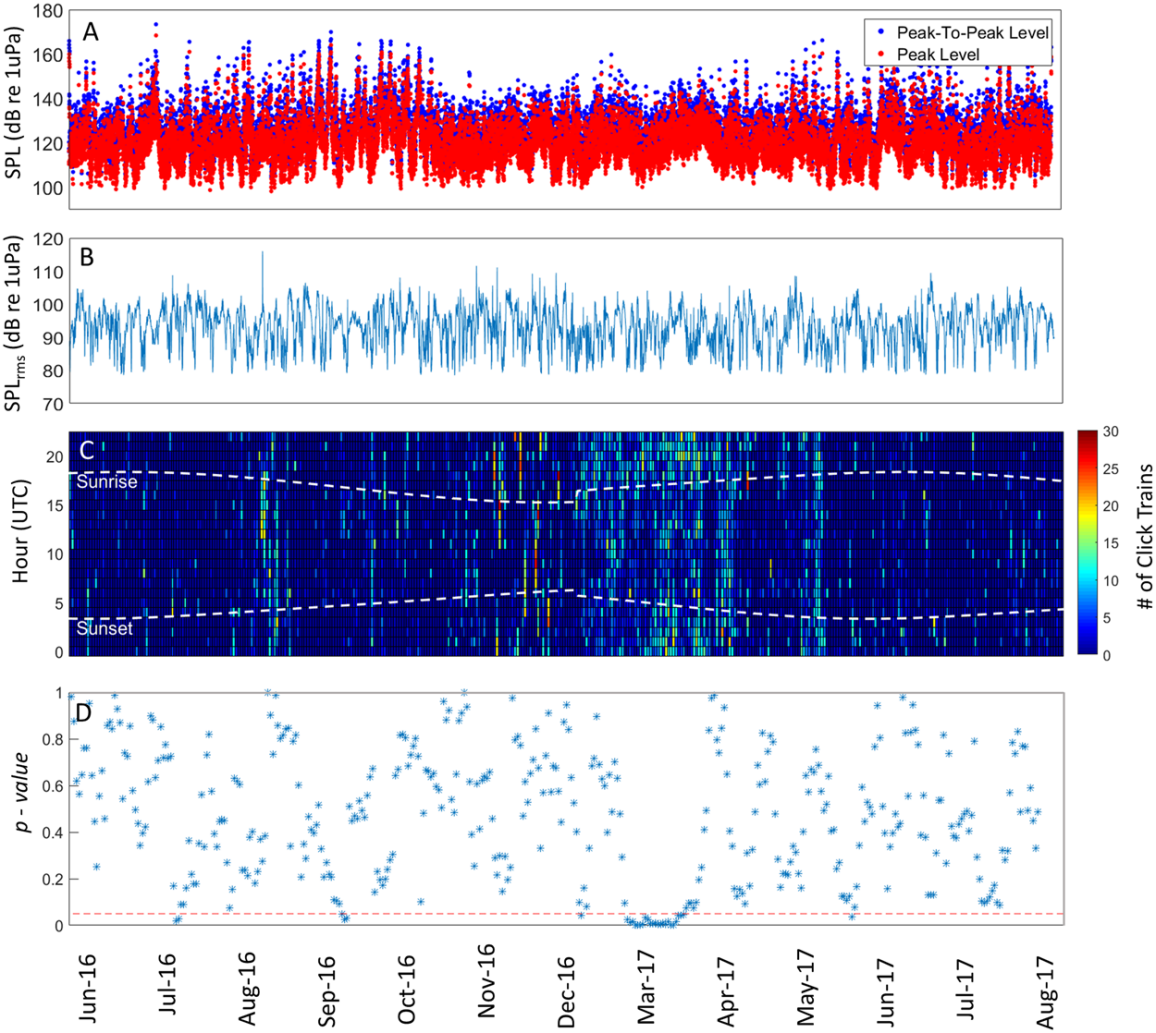
Peak-to-Peak (blue) and Peak (red) Received Levels of the sperm whales click trains detected (panel A); SPL_rms_ Level in the band between 2 and 20 kHz (panel B); Number of click trains detected per hour per day (panel C) and results of the comparison of the number of click trains detected during day-time vs night-time over a 9-day moving window (panel D; one-way ANOVA p-values) for the Palliser station. The white dashed lines in panel C indicate sunset and sunrise times. The red dashed line in panel D indicates the 0.05 significance level of the test. P-values below the red dashed line indicate times when night-time and day-time foraging rates were statistically different. No data were collected between 21^st^ December 2016 and 21^st^ February 2017.

**Figure 4:**
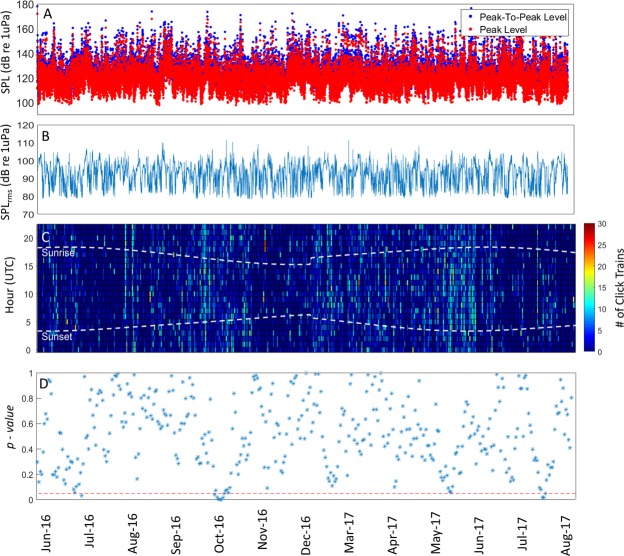
Peak-to-Peak (blue) and Peak (red) Received Levels of the sperm whales click trains detected (panel A); SPL_rms_ Level in the band between 2 and 20 kHz (panel B); Number of click trains detected per hour per day (panel C) and results of the comparison of the number of click trains detected during day-time vs night-time over a 9-day moving window (panel D; one-way ANOVA p-values) for the Kaikoura station. The white dashed lines in panel C indicate sunset and sunrise times. The red dashed line in panel D indicates the 0.05 significance level of the test. P-values below the red dashed line indicate times when night-time and day-time foraging rates were statistically different. No data were collected between 21^st^ December 2016 and 22^nd^ February 2017.

Sperm whales were detected year-round at each station (Figs 2–4, top panels), and the number of click-trains detected ranged between 0 and 30 per hour (median = 4). In general, no statistical difference was observed in click train rates between night-time and day-time at any station (Figs 2–4, bottom panels). Foraging was higher during the day only in March 2017 at the Palliser station (Fig. 3, bottom panel).

**Figure 5:**
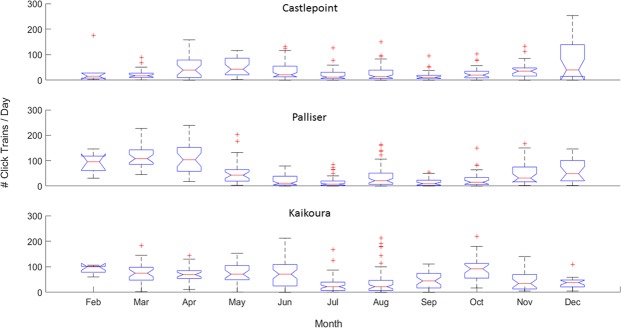
Boxplot of the number of click trains per day detected each month at each station.

**Figure 6:**
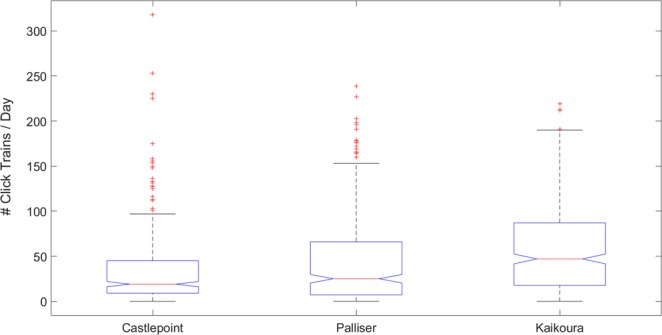
Boxplot of the number of click trains detected per day at each station.

Click trains also varied monthly at all stations (Fig. 5; one-way ANOVA p = 5.38e^−9^ for Castlepoint, p = 3.34e^−41^ for Palliser, and p = 1.31e^−13^ for Kaikoura) indicating that sperm whales may move between stations seasonally. Offshore Castlepoint, click trains were more abundant in April, May, November and December (Fig. 5, top panel). Near Kaikoura (Fig. 5, bottom panel), a decrease in click train detections occurred during the winter months (July and August). The station offshore Palliser showed a cyclical pattern in click train detections, with higher foraging in summer/autumn (~November through April) and lower foraging in winter/spring (~May through October). Overall, the Kaikoura station recorded the highest rate of sperm whale foraging activity (Fig. 6, p = 6.73 e^−11^).

## Discussion

In this study, passive acoustic techniques were used to investigate the foraging activity of sperm whales at three locations on the south-east side of Cook Strait, New Zealand. The foraging dive behavior of sperm whales is well documented and appears to be similar in distinct parts of the world. Data from the Mediterranean Sea and the Gulf of Mexico revealed similar dive patterns with sperm whales diving to depths ranging between 700 and 1100 m^18^, a behavior the was consistent with data from Papua New Guinea^19^, the Atlantic Ocean^17^ and New Zealand^20,21^. The general diving behavior of sperm whale consists in a descent phase (characterized by regular echolocation activity), a bottom phase (usually associated with feeding attempts as evidenced by the production of buzz clicks^18^) and a silent ascent phase^17^. The dive period characterized by regular echolocation activity indicates the time the animal spends searching for prey and it is usually referred to as the “search phase”^17,20^. Hence, by monitoring the echolocation activity using bottom mounted recorders, we are monitoring the “search phase” of sperm whale foraging dives that we can detect at our deployment locations (i.e. the locally detected search phase).

The number of echolocation click trains detected were used as a proxy for foraging activity, and our data does not reflect the abundance of individuals. To account for limited data storage, the recorders were duty cycled (see methods section) to ensure the collection of data throughout each  deployment period at the chosen sampling frequency (250 kHz). The duty cycle (as opposed to continuous recording) might affect the probability of detecting calling whales^22^. Thomisch *et al*.^22^ tested the effects of different duty cycles (against continuous recordings) on the correct estimation of the acoustic presence of whales. The study found that using duty cycles <1/10 reduced the probability of correctly estimate the acoustic presence of calling whales from 1 to ~0.95 in the worst-case scenario (no reduction in probability of correctly estimate the acoustic presence in other cases). For duty cycles >1/10 (as the duty cycle used in our Cook Strait data [equal to 125 s/900 s (0.138)] the probability of correctly estimate the acoustic presence was 1 (or almost 1) in all cases. In the light of Thomisch *et al*.’s^22^ findings, we believe that the duty cycle used in our study is suitable for monitoring extensive periods of time without drastically reducing the probability of correctly estimating the acoustic presence of sperm whales.

Sperm whale foraging activity varied by month at all locations. In particular, the variation we observed in our data at the Palliser station matches the trend described by Gaskin^5^. The whaling catch data indicated a peak in catches between December and April, and a decrease from May to November. The Cook strait sighting data used by Gaskin^5^ were obtained from the whale chaser *Orca*, operated by the Tory channel whaling company, but the precise locations of sighting effort was not reported. It is possible that the main chaser operations were conducted close to Tory channel, so that the sighting data are more representative of the occurrence of sperm whales close to Palliser than areas farther north or south. Additionally, individual sperm whales might have been moving between locations during this study.

Most of the studies conducted on seasonality and abundance of sperm whales in Kaikoura were conducted using sighting data from small vessels. Using a dataset spanning between 1988 and 1993, Childerhouse *et al*.^11^ investigated the abundance and seasonal residence in the Kaikoura area. A total of 86 individual were identified and half of them were sighted over multiple years^11^. However, the distribution of sightings showed differences between summer and winter^12^, with sperm whales more concentrated within the Kaikoura canyon in the summer. These studies evidence a seasonal change in sperm whale habitat use which is further supported by the seasonal variation in foraging activity reported in our data.

Except for a month-long period (March 2017) at the Palliser station, our study found no difference between the night-time and day-time foraging rates of sperm whales. Certainly, the length of the moving window applied to the ANOVA test in our study had an effect on the results. For example, a 50 day-long moving window results in a significant difference (p < 0.05) between Aug-16 and Sep-16 in the Castlepoint data. However, this was an artefact of the window size. When using a long window, large night-time/day-time differences of click trains rates in few single days are accounted for over longer periods of times than when using a short window. In other words, longer windows repeat the ANOVA test over a larger section of the same data. Few of those data were anomalously high (or low) and affected the test results over a longer period. The difference between mid-length windows (20 days) and shorter length windows (9 days) was negligible. Most of the results still indicated no difference between night-time and day-time foraging. We opted for a 9-day window to gain higher temporal resolution of the test.

Although sperm whale diving behavior is somewhat stereotyped, their foraging activity varies in different parts of the world. Off the Kona coast of Hawaii, sperm whales were found to forage more at night only on the northern side of the island^15^. However, on the island of Kauai, within the Hawaiian Island chain, foraging occured mostly at night at different locations around the island^23^, while in the Mediterranean Sea, sperm whale foraging behavior varied seasonally^24^. Along the east coast of the United States, there was no clear diel pattern present in sperm whale foraging activity at five different sites^25^. Differences in sperm whaleforaging activity at different location might have been driven by variation in the abundance and behavior of their local prey. In fact, predators make foraging decisions based on prey distribution gathered from the environment in which they forage. For example, penguins use information from previous dives to plan their next dive^26^. Similar behavior has been observed in cetaceans. Male sperm whales adjust their echolocation behavior at the beginning of a foraging dive to the anticipated range of their prey^27^. Prey behavior has also been shown to affect sperm whale diving behavior. In the Gulf of California, sperm whales preferentially dove within the depthof their local prey, the jumbo squid^28^. In Hawaii, sperm whales seem to forage preferentially in areas where prey density decreases but prey size increases with depth^29^. Hence, from an ecosystem management point of view, it is important to link spatial and temporal variability in prey distribution, to the presence of the predator species. Such knowledge would provide the possibility to rapidly identify potential threats to top predator arising from marine anthropogenic activities in a specific area, and to produce informed ecosystem management decisions.

Similar to other populations, sperm whales in New Zealand feed mainly on squid and fish^30^. Diet studies in New Zealand have been conducted opportunistically via examination of stomach contents of individuals that either stranded on the beach or were caught by whalers. In one study, the ratio of squid to fish (by weight) in sperm whale stomachs was 1.69:1^31^. However, this data might have been biased due to differences in the time prey tissues remain in the stomach of whales. Important prey items in the diet of New Zealand sperm whales include squid families Histioteuthidae, Onychoteuthidae, Cranchiidae, Architeuthidae, Ommastrephidae, and Octopoteuthidae^30,31^ and fish species such as groper (*Polyprion oxygeneios*), ling (*Genypterus blacodes*), orange roughy (*Hoplostethus sp*.) and southern kingfish (*Jordanindia solandri*)^30^. The distribution of two squid species, *Nototodarus sloanii* and *Moroteuthis ingens*, was studied off the South Island of New Zealand^32^, but no seasonality of catches was reported. However, catches per unit effort of Arrow squid’s (genus *Nototodarus*) varied seasonally in many areas around New Zealand^33^. To the best of our knowledge, no seasonal abundance information exists for these squid species near the locations of our study.

The Cook Strait ocean currents are greatly affected by tidal flow. However, residual flow was found to change seasonally, with a northward residual flow forming on the western side of the strait in the summer-autumn period^34^. These changes in flow have substantial implications on the seasonal nutrient supply to the different areas of the strait^34^. How this variability in ocean circulation affects the export of nutrients to the deep ocean and, as consequence, the abundance and distribution of deep sea prey is unknown but may explain the variability observed in the seasonal and spatial foraging of sperm whales.

This long-term passive acoustic monitoring study of sperm whales in New Zealand revealed new insights on their foraging activity. While the Kaikoura canyon is known to be an important foraging ground for this species, our findings demonstrate that other areas of the New Zealand EEZ provide important habitat for  foraging sperm whales year-rounds. A better knowledge of sperm whale foraging activity in various parts of the EEZ is important to understand the habitat use of this predator in New Zealand waters, and to provide useful information for ecosystem management.

## Methods

### Data collection

**Figure 7:**
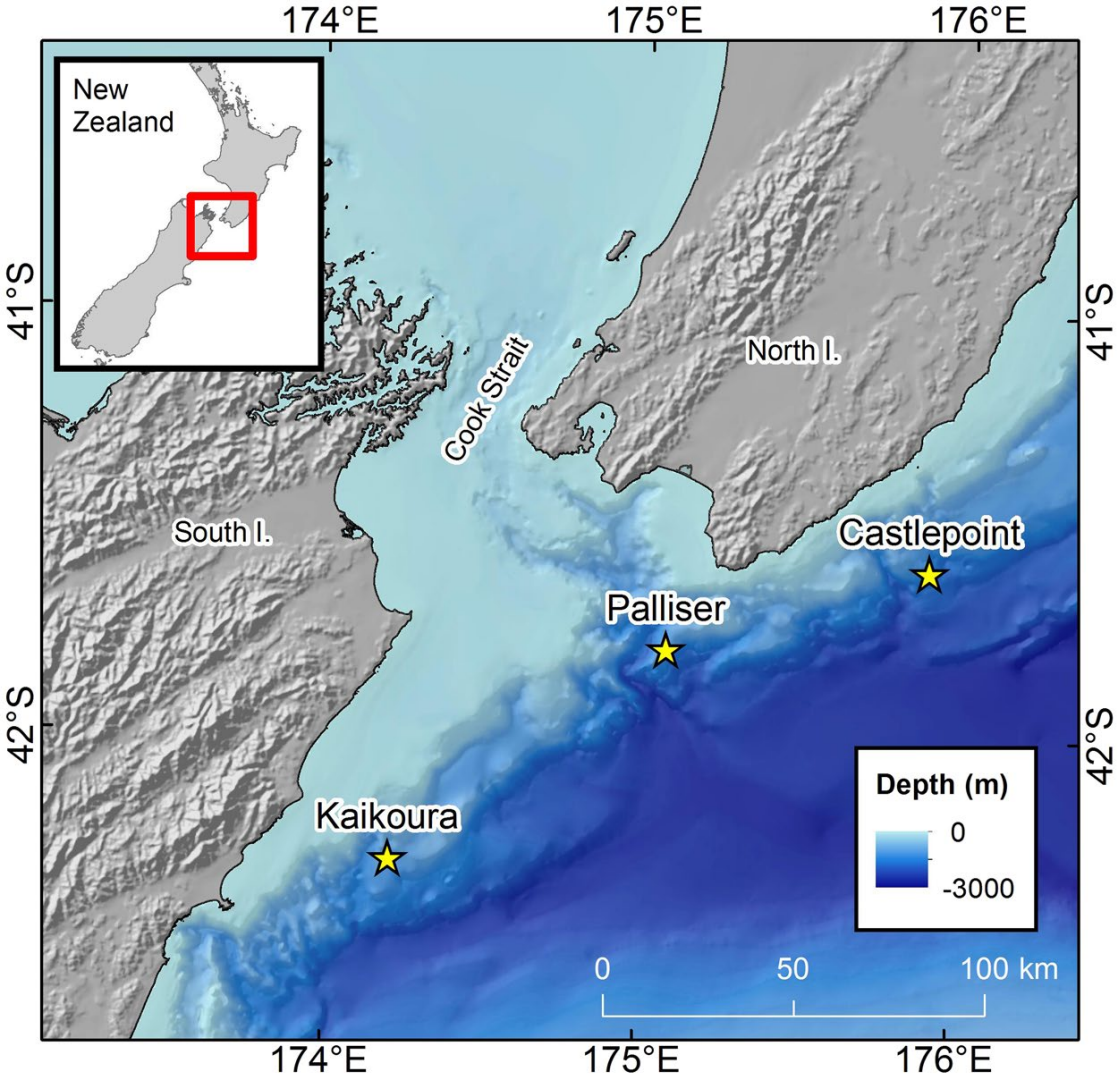
Map of Cook Strait region with the location of the three acoustic moorings indicated by the star symbol. Map created using ArcGIS 10.3.1 (ESRI 2015)^46^.

Three deep-water acoustic moorings were deployed west of the Cook Strait region (>1000 m) in June 2016 (Fig. 7). Each mooring contained an Autonomous Multichannel Acoustic Recorder (AMAR; JASCO Applied Sciences, Dartmouth, Canada) with an M36-V35-100 omnidirectional hydrophone (GeoSpectrum Technologies Inc.; −164 dB re 1 V/μPa sensitivity) and recorded one 125-second-long.wav files at a sampling rate of 250 kHz every 15 minutes. Detailed positions, dates and recording periods of each mooring are reported in Table 1.

**Table 1:** Position and details of the moorings. Dates are reported in UTC time.

Station	Depth (m)	Deployment Date	Re-deployment date	Last Recording	Latitude (Decimal Degrees)	Longitude (Decimal Degrees)
Kaikoura	1250	7 June 2016	22 February 2017	8 September 2017	−42.3087	174.2145
Palliser	1290	6 June 2016	21 February 2017	30 August 2017	−41.8050	175.0810
Castlepoint	1480	6 June 2016	23 February 2017	30 August 2017	−41.6098	175.9029

The recorders were recovered for servicing on 21 December 2016 and re-deployed in February 2017 and finally recovered in August/September 2017. No data were collected between 21^st^ December 2016 and the re-deployment date (Table 1).

### Detection of echolocation signals

Sperm whale echolocation signals were detected and classified using a custom-made algorithm. First, the data were band-pass filtered between 2 and 20 kHz using a sixth order Butterworth filter. The filter cut-off frequencies were chosen according to the bandwidth of sperm whale echolocation signals^35^. The filtered data were then input into the detector. For each.wav file, an adaptive threshold that self-adjusted based on the average ambient background noise level was calculated. The detection threshold was set 4 dB above this average noise level. The algorithm then computed Inter Click Intervals (ICI), duration, and peak frequency of each signal detected above the threshold. The duration of each signal was computed as the time interval between the 5th and 95th percentiles of the signal energy obtained by integrating the square pressure over a 2 ms window around the signal. We selected a 2 ms long window to capture the entire echolocation signal^35^. The echolocation signals detected were clustered into click-trains based on their ICIs. Echolocation signals were grouped in the same click-train if the ICI between consecutive signals was shorter than 3 seconds. This ICI threshold is based on ICI measured in the wild using different acoustic techniques^19,27,36–39^. Following the methods of other studies, only click trains with more than 5 signals were analyzed to strengthen the classification certainty^23,40^. The ICI in the click train would be affected by the presence of multiple sperm whales echolocating at the same time. Since we could not predict the occurrence of multiple individuals, we used a short file length (125-second-long.wav files) to reduce the probability of multiple animals clicking on-axis simultaneously.

The algorithm then computed the probability of each click-train being produced by a sperm whale by assessing whether the peak frequency, ICI and duration of the detected echolocation clicks in the click train fell within a certain range of values. This probability is simply the number of echolocation clicks in the click train that met the requirements for peak frequency, ICI and duration, divided by the total number of echolocation signals in the click train. If the probability was higher than 70%, the click train was considered to be produced by sperm whales.

**Figure 8:**
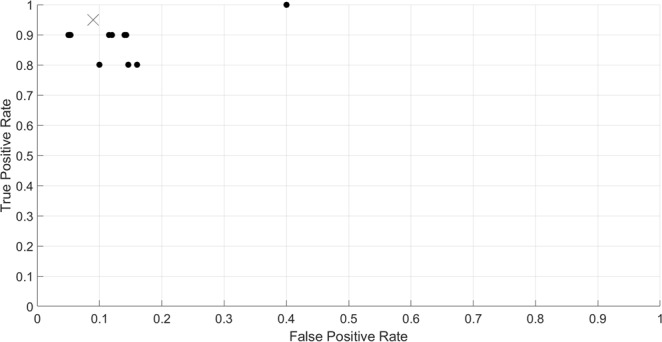
ROC results from the detector training trial (n = 13). Note that plotted data were jittered to enhance plotting clarity. The “x” symbol indicates the trial which range values for peak frequency, ICI and signal duration were selected to be used on the real dataset. The probability of correct response^34,46^ of the trial indicated by the “x” was 0.9.

The ranges for the peak frequency, ICI, and duration ranges were adjusted using a training dataset of 200 .wav files selected among all three stations throughout the deployment. The training dataset was composed as follows: 100 .wav files that did not contain sperm whale echolocation signals (although they did contain different levels of noise from boats, echosounders, and echolocation signals from other odontocete species), and 100.wav files that contain sperm whale echolocation signals (although they also contained different levels of noise from boats, echosounders, multiple sperm whales echolocating at the same times, and echolocation signals from other odontocete species). The best detection/classification performance (over thirteen different trials with varying ranges for peak frequency, ICI and signal duration) was assessed using a Receiver-Operator Characteristic (ROC) approach. The false positive and true positive rates were calculated for each training trial (Fig. 8), and the ranges that yielded false positive and true positive rates as indicated by the “x” sign in Fig. 2 were selected for use. The probability of correct response^41,42^ using such ranges was 0.9. After the training, the selected ranges were 4 to 15 kHz for peak frequency; 0.2 to 2 seconds for ICI; and 500 to 1200 µs for signal duration.

If a .wav file contained a sperm whale click train, then the date and time were logged. This approach of classifying click trains has proven reliable in previous research^15^.

The final output result of the detection algorithm was also compared to the validation computed by an experienced researcher on a subsample of the data. A total of 1200 .wav files (400 randomly selected files for each station) were visually inspected for the presence of sperm whale echolocation signals using spectrograms. The validation results are reported in the result section as precision (P), recall (R) and F-score^43^. P represents the proportion of detections that are true positive, and R represents the proportion of sperm whale echolocation clicks in the dataset that were detected^44^. A β factor of 0.5 was used to assign recall half the weight than precision for the F-score computation^45^.

### Measurements of background noise levels and click trains received levels

The background noise level in the frequency band between 2 and 20 kHz was measured in each.wav file recorded as root-mean-square sound pressure level (SPLrms) in dB re 1µPa using the following equation:

$$SPLrms=20{\mathrm{log}}_{10}\sqrt{\frac{1}{T}{\int }_{T}P{(t)}^{2}dt}$$

where *P(t)* is the acoustic pressure time-series filtered by the same bandpass filter used in the algorithm for the detection of click trains; *T* is the time duration of each.wav file (125 seconds) and *dt* is the time interval between each acoustic pressure measurements.

The received level of click trains was computed as “Peak-To-Peak” and “Peak” Levels (both in dB re 1 µPa) of the loudest click in each click train using the following equations:

$$Peak-To-Peak\,Level=20{\mathrm{log}}_{10}\{max[P(t)]-min[P(T)]\}$$

$$Peak-Level=20{\mathrm{log}}_{10}\{max[|P(t)|]\}$$

where *P(t)* is the 2 ms long acoustic pressure time-series of the selected echolocation click.

### Temporal and spatial analysis

#### Night-time vs Day-time

A one-way ANOVA test applied over a 9-day moving windows was used to compare night-time and day-time foraging activity. The number of click trains detected at night-time and day-time was normalized by the length (in hours) of the night and day respectively, to account for changes in daylight duration between seasons. Hence, foraging activity at night and day are expressed as rates representing the number of click trains per hour. The one-way ANOVA window was set at 9 days after testing the influence of various window lengths on the results. Results were similar for all window sizes shorter than 9 days.

#### Monthly and spatial variation

A one-way ANOVA was also used to compare click trains detections between months and stations. For these two tests, the number of click trains detected per day were grouped by months and stations (no moving window was applied to this test).

## Data Availability

The data generated and analysed during this study are available from the corresponding author on reasonable request at the following web address: http://raven.niwa.co.nz:8080/niwa_dc/srv/eng/main.home.
