# Author Correction: Foraging activity of sperm whales (*Physeter macrocephalus*) off the east coast of New Zealand

**DOI:** 10.1038/s41598-020-58286-y

**Published:** 2020-02-04

**Authors:** Giacomo Giorli, Kimberly T. Goetz

**Affiliations:** 1 National Institute of Water and Atmospheric Research, Coasts and Oceans, 301 Evans Bay Parade, Greta Point, Wellington, 6021 New Zealand; 2 Marine Mammal Laboratory, Alaska Fisheries Science Center, National Marine Fisheries Service, National Oceanic and Atmospheric Administration, 7600 Sand Point Way N.E., Seattle, Washington, 98115-6349 USA

Correction to: *Scientific Reports* 10.1038/s41598-019-48417-5, published online 21 August 2019

In the original version of this Article, Giacomo Giorli was incorrectly affiliated with ‘Marine Mammal Laboratory, Alaska Fisheries Science Center, National Marine Fisheries Service, National Oceanic and Atmospheric Administration, 7600 Sand Point Way N.E., Seattle, Washington, 98115-6349, USA’. The correct affiliation is listed below.

National Institute of Water and Atmospheric Research, Coasts and Oceans, 301 Evans Bay Parade, Greta Point, Wellington 6021, New Zealand

This error has now been corrected in the PDF and HTML versions of the Article.

